# Mitochondrial-Derived Vesicles: The Good, the Bad, and the Ugly

**DOI:** 10.3390/ijms241813835

**Published:** 2023-09-08

**Authors:** Anna Picca, Flora Guerra, Riccardo Calvani, Hélio José Coelho-Júnior, Francesco Landi, Cecilia Bucci, Emanuele Marzetti

**Affiliations:** 1 Department of Medicine and Surgery, LUM University, 70010 Casamassima, Italy; picca@lum.it; 2 Fondazione Policlinico Universitario “Agostino Gemelli” IRCCS, 00168 Rome, Italy; francesco.landi@unicatt.it (F.L.); emanuele.marzetti@policlinicogemelli.it (E.M.); 3 Department of Biological and Environmental Sciences and Technologies, Università del Salento, 73100 Lecce, Italy; flora.guerra@unisalento.it (F.G.); cecilia.bucci@unisalento.it (C.B.); 4 Department of Geriatrics, Orthopedics and Rheumatology, Università Cattolica del Sacro Cuore, 00168 Rome, Italy; coelhojunior@hotmail.com.br

**Keywords:** damage-associated molecular patterns (DAMPs), endosomal–lysosomal system, exosomes, extracellular vesicles, inflammation, mitochondrial quality control, mitochondrial DNA, mitophagy, mitovesicles, oxidative stress

## Abstract

Mitophagy is crucial for maintaining mitochondrial quality. However, its assessment in vivo is challenging. The endosomal–lysosomal system is a more accessible pathway through which subtypes of extracellular vesicles (EVs), which also contain mitochondrial constituents, are released for disposal. The inclusion of mitochondrial components into EVs occurs in the setting of mild mitochondrial damage and during impairment of lysosomal function. By releasing mitochondrial-derived vesicles (MDVs), cells limit the unload of mitochondrial damage-associated molecular patterns with proinflammatory activity. Both positive and negative effects of EVs on recipient cells have been described. Whether this is due to the production of EVs other than those containing mitochondria, such as MDVs, holding specific biological functions is currently unknown. Evidence on the existence of different MDV subtypes has been produced. However, their characterization is not always pursued, which would be relevant to exploring the dynamics of mitochondrial quality control in health and disease. Furthermore, MDV classification may be instrumental in understanding their biological roles and promoting their implementation as biomarkers in clinical studies.

## 1. Introduction

Mitophagy is a major mechanism of mitochondrial quality control. Mitophagy involves the degradation and recycling of damaged or inefficient mitochondria to maintain a healthy pool of organelles and ensure adequate energy provision to cells [1]. However, its assessment in vivo is challenging, which hampers its translational applications [2]. 

The endosomal–lysosomal system encompasses organelles and membranous components that form the endocytic pathway. Through the endosomal–lysosomal system, various cargo molecules are internalized and recycled. Furthermore, this pathway has emerged as a relevant and more accessible component of the mitochondrial quality control system [3,4]. Exosomes, a subtype of extracellular vesicles (EVs) with a diameter of 50–150 nm, are generated and released by the endosomal–lysosomal system. Exosomes are produced from intraluminal vesicles (ILVs), which originate from early endosomes undergoing inward budding of discrete membrane domains that subsequently evolve into multivesicular bodies (MVBs) [5,6,7,8]. MVBs are usually addressed to lysosomes for cargo degradation and recycling. However, under specific stimulation, MVBs can be shuttled toward the plasma membrane for exocytic fusion and subsequent extracellular release of ILVs [5,6]. Under these circumstances, ILVs are called exosomes.

Growing evidence has shown that, depending on the severity of mitochondrial damage, cells can selectively target mitochondrial components for lysosomal degradation and regulate the packaging of mitochondrial constituents into EVs [9]. Mitochondrial disposal via EVs seems to be an alternative strategy to canonical organelle degradation and recycling by mitophagy [9]. The EV pathway of mitochondrial quality control is triggered in the setting of mild mitochondrial damage to avoid wholesale organelle disposal while preventing the release of unpackaged damaged mitochondrial components. The latter would otherwise act as proinflammatory damage-associated molecular patterns (DAMPs) and install an inflammatory milieu [9]. Mitochondrial clearance has also been shown to be accomplished via the endosomal pathway and large EV generation when lysosomal degradation is inhibited [10].

The generation of mitochondrial-derived vesicles (MDVs), small vesicles that shuttle mitochondrial constituents to other organelles, is accomplished through the sorting of mitochondrial components via two different pathways. The first involves the delivery of mitochondrial material to EVs through sorting nexin 9 (SNX9)-dependent MDVs [11]. This vesicle class has been shown to participate in mitochondrial antigen presentation [11]. The second pathway targets MDVs containing damaged mitochondrial components to lysosomes for degradation in a process that is regulated by the mitophagy mediator Parkin [11]. Therefore, the characterization of MDV subtypes represents a unique tool for investigating the dynamics of mitochondrial quality control in health and disease [12]. MDVs also enable intercellular communication with either beneficial or harmful effects on recipient cells, depending on the cellular source as well as the nature of the cargo and the originating stimulus [13]. To further complicate the matter, a distinct population of EVs containing mitochondrial material—mitovesicles—has recently been described and attributed specific signaling functions [14]. All these factors contribute to a limited exploitation of MDVs in the clinical setting, for which a deeper characterization of MDV biology is needed [15].

Herein, we discuss relevant aspects of the trafficking of MDVs, with a focus on their classification and function. We also highlight the importance of a thorough MDV characterization to unveil the biological mechanisms underlying vesicle trafficking in health and disease, and enable their use as biomarkers in clinical studies.

## 2. Mitochondrial-Derived Vesicles

First identified in 2008 by Neuspiel et al. [16], MDVs are single- or double-membrane vesicles, generated, respectively, from the outer mitochondrial membrane (OMM) or inner mitochondrial membrane (IMM) and portions of the mitochondrial matrix. Three independent criteria are used for specifically defining MDVs: (i) generation independent of dynamin-related protein 1 (DRP1); (ii) small size (diameter of 70–150 nm); and (iii) selectivity of the cargo [16,17]. 

Mitochondria have inherited, from their bacterial ancestors, the ability to load their contents into vesicles for long-distance delivery as a constitutive and evolutionarily conserved process [18,19]. These similar prokaryotic vesicles are generated by local evaginations of mitochondrial membranes, vesicle budding, and release into the extracellular compartment. Bacteria release two main types of EVs: outer membrane vesicles (OMVs), generated from the outer plasma membrane layer, and outer–inner membrane vesicles (O–IMVs), containing both outer and inner membrane layer components [20]. OMVs mainly include bacterial lipopolysaccharides (LPSs) from the outer layer, peptidoglycans of the periplasmic space, and unstructured and organelle-free cytosol [21,22]. Conversely, ATP molecules, DNA, and proteins from the cytoplasm and the inner membrane are mostly included in O–IMVs [21,22]. EVs are implicated in multiple bacterial activities, from intracolonial signaling via quorum sensing [23] to intercellular communication via proteins shuttling, as well as in the modulation of immunogenic host invasion, elimination of bacterial competitors, and formation of biofilms [24].

MDVs in eukaryotic cells have more specialized roles compared with their prokaryotic counterparts and have evolved for different purposes [18]. In the setting of early mitochondrial stress, MDVs can shuttle oxidized cargo to lysosomes for degradation, or unload it in the extracellular compartment via EVs, thereby regulating mitochondrial mass more rapidly than mitophagy [3,4,9]. MDVs are highly heterogeneous, and several subtypes have been identified, which also depend on the cell type [25,26,27]. Their heterogeneity is reflected by the multiple processes in which they are involved, including mitochondrial fission, biogenesis of peroxisomes, resistance to oxidative stress and infections, and innate immunity signaling [11,28,29,30,31]. Alterations in the mechanisms generating MDVs have been associated with several pathological conditions, such as neurodegeneration and cardiomyocyte damage under ischemia/hypoxia [4,32,33]. Moreover, altered MDV generation and release have been associated with aging, autoimmune diseases, cancer, and infections [11,34,35]. 

MDV generation is a housekeeping process that occurs at a basal level under physiological conditions [25,36] and is enhanced during pathological stress. An increase in the production of reactive oxygen species (ROS) inflicts damage on proteins, lipids, and nucleic acids, and cells can trigger MDV formation to allow clearance of abnormal mitochondrial particles [28,36,37]. In the setting of mild oxidative stress, the oxidation of mitochondrial membrane proteins initiates the local activation of phosphatase and tensin homologue (PTEN)-induced putative kinase 1 (PINK1)/Parkin, guiding the budding of oxidized membrane proteins and their inclusion into vesicles [4,11]. 

Under physiological conditions, PINK1 is constitutively imported through the mitochondrial import channel of the outer membrane and, when inside the organelle, is cleaved by the protease of the inner membrane (PARL) [38]. Upon cleavage, PINK1 is retro-translocated to the cytoplasm for rapid proteasomal degradation [39], while Parkin resides in the cytosol in the form of an autoinhibited E3 ubiquitin-ligase [33]. Under mild stress, mitochondrial depolarization disrupts the activity of mitochondrial import channels and blocks the internalization of PINK1 that becomes stalled at the import channel or at the outer mitochondrial membrane [40,41]. Here, PINK1 undergoes autophosphorylation and, in turn, phosphorylates the ubiquitin protein and the ubiquitin-like domains of Parkin. These events stabilize Parkin in an active state and facilitate MDV generation and release [40,41]. 

**Figure 1:**
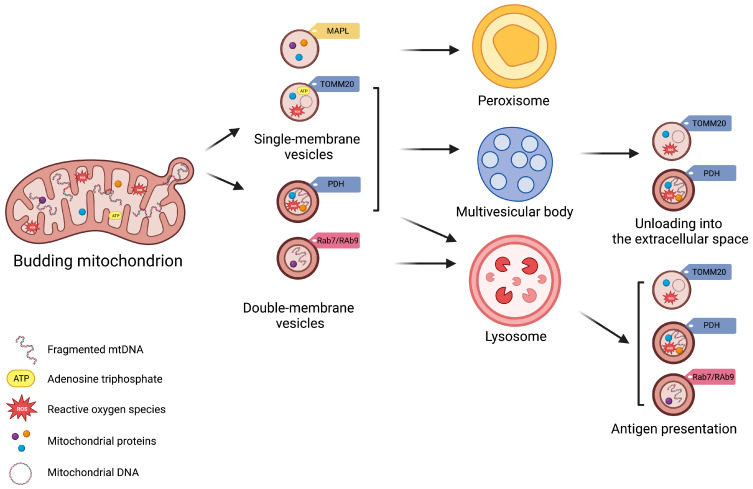
Schematic Representation of Subtypes of Mitochondrial-Derived Vesicles. Mitochondrial-derived vesicles (MDVs) can be classified according to their membrane composition and cargo selection. Based on membrane structure and composition, MDVs can be distinguished as single- or double-membrane vesicles. Single-membrane MDVs incorporate outer mitochondrial membrane proteins, while double-membrane MDVs include outer and inner mitochondrial membrane proteins and constituents of the mitochondrial matrix. According to cargo and membrane protein markers, MDV subtypes include single-membrane MDVs that bear mitochondrial-anchored protein ligase (MAPL) and the import channel translocase of the outer mitochondrial membrane complex subunit 20 (TOMM20), and double-membrane MDVs with pyruvate dehydrogenase (PDH). Vesicle subtypes follow different degradative pathways. Peroxisome is the final destination of single-membrane MDVs with MAPL, while those with TOMM are excreted via multivesicular bodies (MVBs) as exosomes. Double-membrane MDVs with PDH are also released via MVBs. Finally, Ras-related protein (Rab) 7-/9-guided MDV generation mediate mitochondrial antigen presentation via major histocompatibility complex (MHC) class I. Created with BioRender.com, accessed on 3 August 2023.

Matheoud et al. [11] have shown that the biogenesis of MDVs also requires recruitment of the Ras-related protein (Rab9) and SNX9, although the regulation of this process remains unclear. Rab9 is a small GTPase located at the trans-Golgi network and late endosomes, and is implicated in pathways regulating the endo−lysosomal trafficking [42]. Conversely, SNX9 binds directly to the regulator of endocytosis, mediated by the protein clathrin [43]. This pathway generates MDVs for mediating mitochondrial antigen presentation after proteasomal breaking of mitochondrial components into lysosomes and loading of mitochondrial antigens onto major histocompatibility complex class I (MHC I) molecules at the endoplasmic reticulum for their subsequent transfer to cell surface [11,44,45] (Figure 1). Through this pathway, MDVs can regulate survival, development, activation, and differentiation of immune cells (e.g., T-lymphocytes and macrophages) [44,46,47,48]. The destination of MDVs to MVBs for final lysosomal fusion and degradation has also been described as a mechanism to selectively package and dispose mitochondrial constituents via EVs [28,49]. This process prevents the release of oxidized mitochondrial DAMPs with proinflammatory properties [28,49].

Vesicle generation is guided by specific mitochondrial compartments. In particular, β-barrel proteins [50], the mitochondrial-anchored protein ligase (MAPL or Mul1) [51], and the import channel translocase of the outer mitochondrial membrane complex subunit 20 (TOMM20) assist in the formation of single-membrane MDVs [52]. Double-membrane MDVs, instead, are formed by outer and inner mitochondrial membranes that also encapsulate matrix proteins [3,4,16,25,28,53]. These vesicles incorporate specific oxidative phosphorylation (OXPHOS) complexes (e.g., III and IV) of the inner mitochondrial membrane and iron–sulfur clusters to avoid mitochondrial iron overload and remove irreversibly damaged proteins [19]. MDVs may also encapsulate mitochondrial enzymes, including pyruvate dehydrogenase (PDH) and those of the mitochondrial matrix involved in the tricarboxylic acid (TCA) cycle, fatty acids β-oxidation [19], and the antioxidant enzyme superoxide dismutase 2 (SOD2) [28,30]. Among other signaling molecules, mitochondrial DNA (mtDNA) can also be engulfed in MDVs, which has been related to systemic inflammation in several disease conditions [12].

## 3. Mitovesicles

Recently, D’Acunzo et al. [54] published a validated experimental approach to purify and separate different EV subpopulations in vivo from the brain extracellular matrix. These EVs have distinct morphology as well as protein and lipid composition compared with microvesicles and exosomes [54]. The authors coined the term “mitovesicles” to refer to these EVs that bear mitochondrial components including proteins, lipids, and mtDNA [14]. D’Acunzo et al. [54] have established that mitovesicles are distinct from intracellular mitochondria, being 10-fold smaller and having a narrower intermembrane space of about 6 nm, compared with 20 nm in native organelles [55]. 

Mitovesicles also lack several mitochondrial structures, such as cristae, mitochondrial ribosomes, and proteins constitutively found in mitochondria, including the import channel TOMM20 [56]. Interestingly, these vesicles share some features with MDVs described earlier that bud from the mitochondrial surface, including the outer mitochondrial membrane, the inner mitochondrial membrane, and mitochondrial matrix, and are targeted to MVBs [3]. Thus, the possibility that MDVs can be released into the extracellular space as mitovesicles after fusion of MVBs with the plasma membrane cannot be ruled out [57]. However, the mechanisms responsible for mitovesicle generation from mitochondria and their secretion into the extracellular space remain unclear. Moreover, MDVs are heterogeneous, and some of the characteristics of these vesicles are not shared with mitovesicles. Indeed, MAPL, TOMM20, and vacuolar protein sorting-associated protein 35 (VPS35) [3] have not been found in mitovesicles [14]. Furthermore, the tetraspanin CD63, a component of membranes in late endosomes and lysosomes (also indicated as lysosomal associated membrane protein-3 (LAMP-3)), has not been found in mitovesicles, but only in exosomes and MDVs [58,59]. This suggests that distinct mechanisms might be in place to generate different subsets of MDVs with specific functions. One of these subsets would be secreted into the extracellular space, possibly exerting specific extracellular functions rather than serving as mere shuttles of mitochondrial debris outside the cell. In line with this hypothesis, D’Acunzo et al. [14] demonstrated that mitovesicles were not encapsulated by an external membrane, enabling extracellular enzymatic activity of proteins anchored to the outer mitochondrial membrane, such as monoamino oxidase (MAO) A and B, and the capacity to synthesize ATP upon direct contact with adequate substrates. 

A role for mitovesicles has been described for the first time in individuals with Down syndrome (DS), in whom the number of brain mitovesicles is greater than and their composition is different from controls [14]. These findings are in keeping with the observation that EVs of neuronal origin retrieved in plasma of individuals with DS have a different cargo molecule repertoire compared with non-DS controls [60]. Mitochondrial dysfunction, ROS overproduction, and mitophagy deficit have been reported in DS [61]. In this context, mitovesicles, like MDVs, may be part of a mitochondrial quality control pathway that contributes to the removal of detrimental mitochondrial material from the cell and mitigates oxidative stress in a homeostatic feedback loop.

Taken as a whole, changes in intracellular organelle homeostasis may be reflected by different levels of mitovesicles and MDVs, and, more in general, in EV populations such as exosomes [62]. Changes in all these shuttling systems, collectively referred to as EVs, can have pivotal roles as modulators of cell-to-cell communication and in remodeling of the extracellular environment. Whether EVs convey beneficial or negative effects to the recipient cells is discussed in the next sections.

## 4. Mitochondrial-Derived Vesicles and Mitovesicles: Isolation and Characterization Methods

MDVs are a relatively novel aspect of vesicle biology, with limited availability in the literature and knowledge of purification methods. Available techniques for the isolation of secreted MDVs often lead to co-purification of other EV classes, thus making essential their subsequent characterization to distinguish MDV subtypes.

Due to their small size, the isolation of pure EV samples and their quantification are major limitations in the field of EV research. Ultrafiltration, size exclusion chromatography (SEC), precipitation, immunoaffinity-based capture/immunoprecipitation, and ultracentrifugation are current methods for isolation of EV fractions from biological fluids and cellular media [63]. The rapid development of these methods has made isolation procedures easier and faster with larger yields of purified EVs, although disadvantages exist for each of them. 

The ultrafiltration method allows EV concentration on pore-containing membranes and purification based on size. With this method, EV purity is moderate, and membrane clogging and EVs trapped in membranes are common [64]. SEC separates macromolecules based on their size by applying a fluid on a column packed with porous, polymeric beads with the advantage of allowing precise separation of both large and small EVs without affecting their structure. However, this approach requires a long runtime [65]. The precipitation method is an easier procedure, but it determines alteration of EV solubility or dispersibility with possible co-precipitation non-EV contaminants, such as proteins and/or polymeric materials [66]. Finally, the immunoaffinity-based capture/immunoprecipitation based on specific interactions between a membrane-bound antigen of EVs and an immobilized antibody allows for isolating highly purified EV fractions with the possibility of subtyping. However, this method requires knowledge of EV tags, and co-purification of different EV subpopulations sharing similar EV tags may occur [67]. 

The ultracentrifugation method remains the elective approach for the purification of EVs as recommended by the International Society of Extracellular Vesicles (ISEV) [68]. The method is based on density-, size-, and shape-based sequential separation, consisting of several centrifugation steps to remove cells, debris, and, finally, pelleted EVs. This approach is the most used preparative procedure for EV purification and has also been applied for MDV characterization [57]. Via ultracentrifugation, it is possible to obtain a good yield of EVs/MDVs starting from different matrices, such as cell media, plasma, serum, or urine samples. However, there are, also in this case, some caveats. Ultracentrifugation allows purification of heterogenous classes of EVs, among which MDVs are present. The isolation of one type of EV is unfeasible with this method and, after ultracentrifugation, other methods become essential for MDV characterization [3,10,57,69,70]. As per our protocol of MDV/EV purification starting from serum/plasma, samples are diluted with equal volumes of phosphate-buffered saline (PBS) before centrifugation to reduce fluid viscosity and subjected to a first centrifugation at 2000× *g* at 4 °C for 30 min to discard any cell contamination [57]. Supernatants are then centrifuged at 12,000× *g* at 4 °C for 45 min to remove apoptotic bodies, cell debris, and large vesicles (mean size > 200 nm) and, subsequently, ultracentrifuged at 110,000× *g* at 4 °C for 2 h. Pellets are recovered and resuspended in PBS, filtered through a 0.22 μm filter, and ultracentrifuged at 110,000× *g* at 4 °C for 70 min to eliminate contaminant proteins. Pellets enriched in purified EVs, among which MDVs are also included, are resuspended in 100 μL of PBS. To quantify EVs, total protein concentration is measured using the high-sensitivity BCA assay [57]. 

According to ISEV guidelines, purification methods should be validated by complementary approaches such as electron microscopy analysis (TEM), nanoparticle tracking analysis (NTA), and Western immunoblotting. The latter technique is useful not only to verify the occurrence of positive and negative markers of purification, but also to characterize the protein content of EVs and determine whether the purified populations also contain MDVs [3,10,57,69,70]. This analysis can be performed using specific antibodies that recognize MDV markers such as TOMM20, ATP synthase subunit 5A (ATP5A), mitochondrial cytochrome C oxidase subunit I (MTCOI), NADH:ubiquinone oxidoreductase subunit B8 (NDUFB8), NADH:ubiquinone oxidoreductase core subunit (NDUFS3), succinate dehydrogenase complex flavoprotein subunit A (SDHA), succinate dehydrogenase complex iron–sulfur subunit B (SDHB) and, ubiquinol–cytochrome C reductase core protein 2 (UQCRC2) [57,69,70]. Further confirmation of mitochondrial origin can be obtained via the identification of mtDNA encapsulated within these vesicles [71].

Alternatively, MDVs can be visualized as small vesicular structures that show cargo selectivity under microscopy approaches when using highly specific antibodies against endogenous mitochondrial proteins or a combination of transfected mitochondrial green fluorescent protein (GFP)-tagged constructs with antibodies to label a second or third mitochondrial protein [3]. The absolute dependence on protein specificity and background signals are important limitations related to the use of antibodies. In addition, GFP-tagged and overexpressed proteins are not always efficient for cargo selectivity, perhaps because they are first targeted by proteases. The analyses of mitochondrial proteins and mtDNA content in MDVs, as well as proteomic approaches, remain more relevant analytical tools. 

Ultracentrifugation is also a preparative method for subsequent isolation of mitovesicles using a high-resolution density gradient [14,54]. By slight modification of the original sucrose gradient isolation method, D’Acunzo et al. [14,54] isolated and fractioned EVs with an iodixanol-based step gradient density column. They were the first group to develop a method to isolate EVs from murine and human postmortem brains using a sucrose-based step gradient [54,72,73,74]. The method involves a short enzymatic digestion of the brain tissue to loosen the extracellular matrix (ECM), followed by differential centrifugation and a sucrose gradient [75,76]. Because the sucrose-based gradient is hyperosmotic compared with biofluids, this method causes vesicle shrinkage, which may be of concern if biologically active EVs are required for later analyses. Furthermore, it is impossible to generate high-resolution step gradients with sucrose as a density medium with sucrose-based solutions with similar molarities, making the separation of EV subpopulations difficult. As a consequence, the authors modified the fractionation method by using iodixanol to create the density column, demonstrating a successful separation between different subtypes of brain EVs [14,54]. Iodixanol is isosmolar with body fluids across a wide range of dilutions, is inert, has relatively low viscosity, and allows generation of fractions with closer density ranges than sucrose, enabling efficient EV separation with higher resolution power. After enzymatic digestion of tissue, the EV pellet is purified using the ultracentrifugation method described above and, after stratification on iodixanol gradient, fractions containing microvesicles, exosomes, and mitovesicles are obtained. According to ISEV recommendations, the latter can be further analyzed by NTA, electron microscopy, Western blot analysis, and measurement of microvesicular ATP kinetics [14,54].

In conclusion, MDVs can be purified by using several approaches. As for the currently available methodologies, secreted MDVs can be characterized on the basis of their content and quantified by measuring mitochondrial protein and mtDNA amounts.

## 5. Mitochondrial-Derived Vesicles: The Good

The maintenance of a healthy and fully functional population of mitochondria requires the clearance of dysfunctional and/or damaged organelles. In eukaryotic cells, a quality control system is in place that involves several pathways distinctly activated according to the nature and severity of mitochondrial dysfunction [1]. In this function lies the first “good” role of MDVs. Indeed, MDVs have been defined as the first line of defense against mitochondrial stressors due to their ability to remove oxidized mitochondrial components, avoiding complete disposal of organelles by mitophagy [19,25,30,32,53,77]. MDVs allow preservation of the mitochondrial proteome (>1000 proteins) and a functional integration of mitochondrial activities according to the cellular energy requirements [19,25,30,32,53,77]. For instance, in cancer cells, but also in other cell types with inefficient mitophagy, the generation of MDVs and the subsequent lysosomal elimination of damaged mitochondrial particles operate as a compensatory, adaptive mechanism to support mitochondrial health [78,79,80]. For the same reason, such as their ability to degrade damaged cargo, MDVs are responsible for mitochondrial turnover and organelle renewal with functional proteins and lipids. 

MDVs are also pivotal in mediating inter-organellar communication, which represents an additional evolutionarily conserved mitochondrial ancestry [16,18,19,30,77]. Studies have shown the ability of mitochondrial ancestors to release vesicles containing specific molecules with multiple functions, including transportation of virulence factors, delivery of antigens, and interbacterial communication [24]. Among MDV-inherited ancestry functions are their anti-apoptotic and antimicrobial effects. For instance, MDV release is enhanced by hypoxia, during which encapsulation and transfer of B-cell lymphoma 2 (BCL-2) factors blunt mitochondrial-mediated apoptosis and alleviate myocardial ischemia [32]. Similarly, SOD2 is shuttled between mitochondria and phagosomes as an MDV cargo to enhance its antimicrobial effect [30], and MAPL is translocated between mitochondria and peroxisomes for the biogenesis of pre-peroxisomes [81]. In this case, MDVs are also implicated in the de novo biogenesis of peroxisomes [16,82]. MDVs containing the E3 ubiquitin ligase MAPL peroxisome marker can be generated by either the growth and division of pre-existing organelles or de novo biogenesis [82]. Although this latter case has been extensively documented in yeasts, fewer studies have described this process in mammalian cells. Recently, it has been demonstrated that the fusion of vesicles containing proteins involved in the initiation of peroxisome biogenesis, referred to as “peroxins” (Pex) [16,82], triggers the generation of immature pre-peroxisomes. Two distinct organelles are involved in creating the vesicles that participate in the fusion process: (i) mitochondria, with Pex3-rich MDVs (generated from the outer mitochondrial membrane)/Pex14, the integral protein membrane responsible for the import of peroxisomal matrix, and (ii) the endoplasmic reticulum, contributing Pex16-rich vesicles. The resulting fused structure imports proteins of the peroxisomal membrane into the lipid bilayer through the mediation of Pex3 and Pex16, and recruits proteins for the matrix from the cytosol [82]. Upon import completion, fully competent and mature peroxisomes grow, elongate, and divide into daughter organelles, and regulate their abundance according to cellular needs [16,82].

MDVs have also been involved in antimicrobial defense by converging to bacteria-containing phagosomes [83]. For instance, it has been shown that the infection of macrophages with methicillin-resistant *Staphylococcus aureus* (MRSA) triggers the generation of MDVs loaded with SOD2 [30]. Once generated, these vesicles can be delivered to the bacteria-containing phagosomes, whereby the SOD2 antioxidant cargo converts superoxide anions (O^−^⋅) into hydrogen peroxide (H_2_O_2_). These latter molecules are used to kill the invading bacteria [83]. Finally, MDVs can also convey “good” signals in the settings of different disease conditions (Table 1). Indeed, these vesicles exert different roles in myocardial ischemia, neurodegenerative diseases, skeletal myocytes, liver, brown adipose tissue, and cancer cell metabolism [3,32,50,69,70,79,84,85,86]. Mitochondrial homeostasis and innate immune signaling favoring muscle remodeling are also ensured by MDVs in skeletal myocytes [87]. MDVs have been shown to have protective roles against liver injury induced by chronic alcohol exposure [84]. Of note, these vesicles have not been identified in the liver of Parkin knock-out mice [85]. Recently, MDV release has also been indicated as a biomarker of liver disease [88]. The intramyocardial injection of MDVs released by stem cell-derived cardiomyocytes has been shown to restore the bioenergetic failure of cardiomyocytes after myocardial infarction [89].

Several studies have also reported positive roles for the horizontal transfer of EVs that bear mitochondrial components. This transfer can be extended to distant cells/tissue, as shown for EVs carrying oxidized mitochondrial components released by palmitate-stressed adipocytes [90]. In vivo, these vesicles can be taken up by cardiomyocytes and trigger ROS production [90]. This horizontal mitochondrial transfer may serve as a protective preconditioning signal against myocardial injury [90]. The injection of EVs obtained from energetically stressed adipocytes from mice prior to coronary artery ligation alleviated cardiac ischemia/reperfusion injury [90]. This cardioprotective effect was absent in Parkin knock-out mice with adipocytes producing lower mitochondrial-enriched EVs [90]. Thermogenically stressed brown adipocytes also secrete EVs containing oxidized mitochondrial components that can undergo re-uptake by parental brown adipocytes, reducing protein levels of the peroxisome proliferator-activated receptor gamma (PPAR-γ) and the uncoupling protein 1 (UCP1) in recipient cells [86]. The removal of these vesicles by brown adipose tissue (BAT)-resident macrophages via phagocytosis is deemed to be necessary for maintaining tissue homeostasis [86]. A similar mechanism was also described in the heart of mice in which cardiac resident macrophages execute phagocytosis of EVs that contain mitochondria released by cardiomyocytes [91]. This is accomplished through the recognition of a phosphatidylserine residue on the EV surface by the macrophage receptor tyrosin–protein kinase Mer, mediating phagocytic uptake [91]. Mice under catecholamine or coronary artery ligation stress showed higher phagocytic removal of cardiomyocyte-released EV-containing mitochondria [91]. Moreover, the depletion of cardiac resident macrophages in these mice was associated with diastolic dysfunction and reduced survival after coronary artery ligation [91]. 

A significant enrichment of mitochondrial proteins as well as entire mitochondria with intact membrane potential and respiration has also been found in EVs released by neural stem cells (NSCs) [92]. The addition of these EVs to L929 Rho0 cells lacking mtDNA restored mitochondrial activity and increased cell viability [92]. Moreover, the integration of EV-contained mitochondria into mononuclear phagocytes cells re-established metabolism and mitochondrial dynamics and attenuated the expression of proinflammatory markers [92]. The injection of exogenous NSC-released EVs into animal models of multiple sclerosis improved neuroinflammation [92]. In addition, mitochondrial transfer via large-microvesicle EV fraction in cell models of cerebral ischemia significantly increased ATP levels in recipient cells and increased cell survival [93].

Finally, electron microscopy analyses have revealed that human renal carcinoma cells are able to release MDVs and endoplasmic reticulum-derived vesicles [94]. In cancer cells, the rare autophagy-deficient clones are characterized by an increase in MDV levels [79], favoring the hypothesis of this route being an alternative mitochondrial quality control pathway to canonical mitophagy.

**Table 1:** Studies indicating positive effects conveyed by mitochondrial-derived vesicle signaling in several conditions.

Condition	Biological Mechanism	Reference
Doxorubicin-induced cardiotoxicity	Clearance of damaged mitochondria via MDV release in doxorubicin-induced mitochondrial and cardiac toxicity	[25]
MRSA infection	MDV-guided ROS delivery into bacterial-containing phagosomes improves macrophage antimicrobial function	[30]
Cardiac hypoxia	MDV delivery blunts hypoxia-induced cardiomyocyte apoptosis via BCL-2 signaling	[32]
Cancer	Autophagy-deficient cancer cell clones increase mitochondrial dynamics and MDV-mediated mitochondrial recycling to compensate for loss of canonical autophagy	[80]
Parkinson’s disease	High serum level of MDVs associated with specific inflammatory molecules	[70]
Physical frailty and sarcopenia	High serum level of MDVs associated with specific inflammatory molecules	[69]
Alcohol-induced liver injury	Mitophagosomes formation in hepatocytes of rats under chronic ethanol treatment	[84,85]
Myocardial infarction	MDVs generated from autologous cardiac stem cell restore mitochondrial bioenergetics of cardiomyocytes after myocardial infarction	[89]
Renal carcinoma	Electron microscopy observation of vesicular structures derived from the endoplasmic reticulum or mitochondria with unclear function	[94]

Abbreviations: BCL-2, B-cell lymphoma 2; MDVs, mitochondrial-derived vesicles; MRSA, methicillin-resistant *Staphylococcus aureus*; ROS, reactive oxygen species.

## 6. Mitochondrial-Derived Vesicles: The Bad 

A major beneficial role of MDVs has been identified in the mediation of antigen presentation for the regulation of immune responses [11,44,45]. As previously described, this process requires Rab9 and SNX9 proteins, whose mitochondrial recruitments are inhibited by the mitophagy mediators PINK1 and Parkin in Parkinson’s disease (PD) [11]. 

While supporting the generation of MDVs as an alternative route to mitophagy, this also indicates a potential role of the mitophagy mediators PINK1 and Parkin as suppressors of the innate immune response in PD by blunting antigen presentation [11]. Mitochondrial vesicles have also been implicated in pathophysiological signaling [46,95] or, at least, to hold different cargo types and levels, depending on the disease condition [14]. A “negative” role has been attributed to MDVs that seems to be conveyed mainly by responses that mount following cell/tissue injury through the release and recognition of mtDNA as part of DAMPs (Table 2). 

Cell-free/vesicular mtDNA can bind to intracellular Toll-like receptors (TLRs) or nucleotide-binding oligomerization domain-containing protein (NOD)-like receptors (NLRs) to modulate innate immunity and associated inflammation [96]. At the extracellular level, instead, mitochondrial DAMPs can bind to pattern recognition receptors (PRRs) and further ignite tissue and organ injury via inflammatory signaling [9,96,97,98,99]. Indeed, mitochondrial DAMPs can guide the migration and degranulation of neutrophils at the site of injury, thereby further promoting cellular damage and local inflammation [9,96]. In addition to mtDNA, ROS release by mitochondria has been shown to trigger proinflammatory signaling by enhancing the expression of the nuclear factor kappa B (NF-κB) gene and promoting hypoxia-inducible factor 1α (HIF1α)-induced formation of the NLR family pyrin domain-containing 3 (NLRP3) inflammasome [100,101].

The release of mtDNA through MDVs generated via the SNX9 pathway has been documented in adult mice bearing mutations in fumarate hydratase (FH) [102]. mtDNA unloading in cytosol in the setting of FH loss has been associated with altered mitochondrial morphology. Once released, mtDNA triggers innate immunity via the cyclic GMP–AMP synthase (cGAS)-stimulator of interferon genes (STING)–TANK-binding kinase 1 (TBK1) pathway [102]. The ensuing inflammatory response has also been shown to depend, at least partly, on the mitochondrial retinoic acid-inducible gene I (RIG-I) signaling pathway [102]. mtDNA transfer via EVs has also been described in xenograft models of hormonal therapy-resistant metastatic breast cancer and cancer-associated fibroblasts obtained from patients [71]. mtDNA shuttled within EVs conveys oncogenic signals that promote the exit from dormancy of therapy-induced cancer stem-like cells, which has been associated with resistance to therapy in OXPHOS-dependent breast cancer cells [71].

MDV-associated proinflammatory effects not mediated by mtDNA have also been described. Microvesicle-enriched mitochondria generated by LPS-treated monocytes activate endothelial cells and trigger inflammation [103]. Conversely, exosome-enriched mitochondria do not show proinflammatory properties [9]. Indeed, macrophage-triggered interleukin 6 (IL6) secretion occurred in response to mitochondria exposure but not to exosomes isolated from mouse embryonic fibroblasts [9].

Although no mechanistic conclusions can be drawn, a relationship between MDVs and inflammatory mediators has been described in human age-associated conditions, including PD and physical frailty and sarcopenia (PF&S) [69,70]. In these conditions, higher levels of extracellular vesicles of endosomal origins were observed, while lower levels of mitochondrial electron transport chain constituents were identified as part of EV cargo [69,70]. The apparent reduced activity of the mitophagy apparatus in the setting of PD and PF&S can explain, at least partly, the high levels of EVs trying to extrude damaged organelle constituents (which triggers inflammation). However, MDV release can also quench inflammation and the downstream inflammatory cascade by delivering mitochondrial components to lysosomes for degradation and fusion with MVBs [104,105]. EV-mediated quenching of inflammation has also been reported in macrophages taking up mitochondria via EVs produced by mesenchymal stem cells (MSCs) [106]. An inhibition of TLR-mediated inflammation was observed, which occurred through the production of exosomes containing micro-RNA by MSCs that desensitized macrophages by inhibiting TLR [106]. This process may be in place to circumvent the possibility of blunting mitochondrial transfer under proinflammatory signals generated by mitochondrial release. A thorough characterization of these vesicles is needed in terms of membrane composition and cargo nature before solid conclusions can be drawn. 

**Table 2:** Studies reporting negative effects conveyed by mitochondrial-derived vesicles signaling in several conditions.

Condition	Biological Mechanism	Reference
Cell injury	High mtDAMPs and inflammation	[96]
Trauma and post-injury sepsis	High mtDAMPs and inflammation	[97]
Chronic inflammation	High mtDAMPs and inflammation	[98]
SIRS and MODS	High mtDAMPs and inflammation	[99]
Innate immunity activation	Vesicular release of mtDNA	[102]
Breast cancer	mtDNA transfer via exosomes and escape from dormancy of hormonal therapy-resistant breast cancer cells	[71]

Abbreviations: MODS, multiple organ dysfunction syndrome; mtDAMPs, mitochondrial damage-associated molecular patters; mtDNA, mitochondrial DNA; SIRS, systemic inflammatory response syndrome.

## 7. Mitochondrial-Derived Vesicles: The Ugly

As mentioned earlier, mitochondria also regulate cellular metabolism by interacting physically and functionally with lysosomes. However, the exact mechanisms of mitochondria–lysosome communication as well as their upstream signaling and biological functions remain unclear. Mitochondrial remodeling via inter-organelle contacts and fusion during hypoxia allows generation of organelles ranging in shape from tubular to enlarged, dysmorphic, “ugly” megamitochondria [107]. Mitochondria–lysosome contact enables the engulfment of lysosomes by megamitochondria in a process termed megamitochondria engulfing lysosome (MMEL) [107]. MMEL requires the co-existence of megamitochondria and mature lysosomes, in addition to a set of protein mediators, including STX17, synaptosome-associated protein 29 (SNAP29), and vesicle-associated membrane protein 7 (VAMP7), that assist in the formation of a mitochondria–lysosome complex [107]. The recruitment of lysosomes to megamitochondria, followed by mitochondrial clearance, has been proposed as a process of mitochondrial self-digestion for quality control purposes [107].

Evidence has also been produced on noncanonical outsourcing mechanisms for organelle clearance via unknown signaling roles. The generation of extracellular particles containing mitochondria, called exospheres, via membrane blebbing has been described above as part of the mechanisms that mediate mitochondrial horizontal transfer with a role in preserving tissue homeostasis [86,91,108]. Via similar mechanisms, neurons from *C. elegans* extrude exospheres containing protein aggregates and organelles for cell quality purposes under neurotoxic conditions [109]. The actual pathophysiological implications of MMEL and its occurrence in settings other than hypoxia need to be ascertained. Likewise, mitochondrial horizontal transfer systems as additional noncanonical routes of mitochondrial quality control and their involvement in health and disease are worth exploration.

## 8. Therapeutic Applications of Mitochondrial Transfer

Mitochondrial transplantation has gained attention as a therapeutic strategy for bioenergetic reprogramming and organelle trafficking [110]. The regenerative ability of isolated mitochondria in myocardial ischemia has been widely investigated. The first evidence, obtained in 2009, showed that the injection of autologous respiration-competent mitochondria isolated from non-ischemic heart zones into heart ischemic areas before reperfusion reduced infarct size and increased cell viability in rabbits [111]. Subsequent larger animal studies confirmed these initial findings [112,113]. Some years later, in 2017, the first clinical trial on autologous mitochondrial transplantation was carried out in pediatric patients who required extracorporeal membrane oxygenation after ischemia−reperfusion injury [114]. Autologous mitochondria isolated from skeletal muscle biopsies were injected intramyocardially during the surgical procedure [114]. Additional clinical trials are currently underway to test the feasibility of mitochondrial transplantation as a treatment in different settings (NCT02586298, NCT02851758, NCT04998357, NCT04976140). 

However, a major limitation of these methods, especially those involving myocardial injection, lies in the fact that they require surgical access and multiple administrations [112,113,115]. Therefore, devising minimally invasive approaches is highly sought after. Recently, the administration of intermyofibrillar mitochondria isolated from murine skeletal muscle to myoblasts resulted in time- and dose-dependent incorporation of mitochondria and organelle elongation, as well as improvement in myoblast bioenergetics [116]. The transfer of murine muscle mitochondria was also accomplished in human fibroblast bearing mtDNA mutations, with the effect of promoting mitochondrial dynamics and metabolism while reducing ROS levels [116]. Mitochondrial transfer occurred via EVs, gap junctions, micropinocytosis, and tunneling nanotubes, thus holding promise for less invasive strategies to be further developed through EV exploitation [116].

## 9. Conclusions and Perspectives

A role for MDVs as indicators of endo–lysosomal activity in the context of mitochondrial quality control has increasingly been recognized. While studies indicate the potential role of MDVs as biomarkers of disease progression and promising tools for developing innovative approaches to rescue mitochondrial failure [92,116], many of these findings were obtained in preclinical models and several aspects of MDV biology remain undeciphered. These include the classification of MDV subtypes, the definition of their functions, the dissection of the signaling pathways involved, and their characterization in human diseases. This knowledge would shed light on the molecular mechanisms underlying MDV trafficking in health and disease, and may allow their implementation as biomarkers in clinical studies.

## Data Availability

Not applicable.
